# Epidemiological Study of Youth Mental Health Using Strengths and Difficulties Questionnaire (SDQ)

**Published:** 2012-06-30

**Authors:** S Arman, M Keypour, M R Maracy, A Attari

**Affiliations:** 1 Behavioral Science Research Center, Isfahan University of Medical Sciences, Isfahan, Iran; 2 Department of Community Medicine, Isfahan University of Medical Sciences, Isfahan, Iran

**Keywords:** Youth, Strength and difficulties Questionnaire, SDQ, Mental health, Relational problem, Conduct problem

## Abstract

**Background:**

Prevention and treatment of youths` mental disorders are important, not just for to diminish their present problems but to improve their functions in adulthood. This study was performed to evaluate the mental health status of 6-18 years old youths in a community-based sample using the strength and difficulties questionnaire (SDQ).

**Methods:**

Parent of 2000 youths (6-18 years old) were selected from 250 clusters from different municipality areas of Isfahan, Iran and they responded to extended version of SDQ parent`s form. The mean score in each subscale and prevalence of symptom and relation with some demographic features were determined.

**Results:**

The total score of SDQ was 26%, means that 26% of youth had psychiatric problems. By determining the subscale score, it reveals that conduct disorder (34.7%) was the most common problem in youth followed by peer relationship problems (25.4%), emotional problems (24.5%), hyperactivity (23%) and impairment of prosocial behavior (5.7%).

**Conclusion:**

Our findings showed that mental health of youth population in Isfahan especially the conduct problem needs more attention.

## Introduction

Mental disorders are among common health problems all over the world.[1] Socioeconomic, political and cultural changes may affect the physical, mental health and life style of youths.[2] Behavioral and emotional problems in youth may cause a noticeable distress not just among them but in their families too.[3][4]

Prevention and treatment of youths` mental disorders are important, not just to diminish their present problems but to improve their functions in adulthood.[5] Psychiatric disorders have a high prevalence among youth[5] and could be accompanied with other problems including poor school performance, chronic health problems[6] or intensifying the present medical problems,[7] substance abuse and suicidal behaviors.[6] Studies about prevalence of psychiatric disorders among youth population in various geographic places of the world are different with a rate of 10% in developed countries and 15% in developing countries.[8]

In a Siberian study in 2007, the prevalence of psychiatric disorder in youths was reported about 15-20%.[9] In a study in Norway (2004), they reported that one third of children had minor problems and 5% had major or severe psychiatric disorders.[10]

Simpson et al. in USA reported that 5% of children aged 7-14 years suffered from emotional and behavioral disorders that affect their functioning, learning, friendship, family life and joyful activities.[3]

Iran is a developing country whose rapid socioeconomic changes could affect children and adolescents` mental health. According to the recent census, 20% of Iran population is under age of 20 years but there is not any precise estimation about youth psychiatric problems in Iran and the recent researches have been carried out in low number of samples.[11]

One study in Rafsanjan, Iran on students aged 15-18 years reported that prevalence of all psychiatric disorders was 16.6%.[12] In another study among students aged 7-11 years in Isfahan, prevalence of behavioral disorders was estimated 15.5% and 29.8% by using Rutter and CSI-4 questionnaires respectively.[13] The present study was designed to evaluate the frequency of psychiatric disorders in a community sample of 6-18 years old from different municipality areas of Isfahan.

## Materials and Methods

This is a cross-sectional study carried out in Isfahan, Iran in 2008. The population comprised youths aged 6-18 years whose parents were questioned. The sampling method was a random multistage cluster one based on population distribution (250 boys and 250 girls from each of age range of 6-9, 10-12, 13-15 and 16-18 years) with a sample size of 2000.

The examiner team referred to sample's address and after giving explanation to them about the research and obtaining their consent, SDQ parent report form as well as form of demographic information were completed. SDQ questionnaire was a structured questionnaire to screen psychiatric problems of youths containing 25 questions and 5 subscales including emotional, hyperactivity, peer relationship, conduct problems and prosocial behaviors. There were 5 items for each subscale. Each item was given a score (0-2) and the total score of each subscale was considered from 0 to 10 and classified as normal, borderline and abnormal. Total difficulty score was calculated by adding the four first subscales ranking from 0 to 40. The cut of point of SDQ was regarded 17 for children and 20 for adolescents.[14][15]

The questionnaire contained parent, teacher and self reports.[16] Psychometric property of the Persian version of SDQ was provided and its reliability and validity were acceptable.[14] The internal reliability testing of the total SDQ showed a Cronbach`s alpha of 0.73.[14][16] The extended version of SDQ form was used in the present study contained impact score and had items on overall distress and social impairment (score of 2 or more was considered abnormal).[15]

The data was entered to SPSS (version 18, Chi-cago, IL, USA) software and was analyzed by descriptive statistics (frequency distribution, mean and SD) and analytical statistics of multinominal logistic regression test. A p value less than 0.05 was considered significant.

## Results

Among the study population, 513 samples (26%) had a total problem score higher than cut of point, with no significant differences in both sexes. Regarding subscales of disorders, conduct disorder had the highest prevalence (34.7%) followed by peer relationship problem (25.4%), emotional problem (24.5%), hyperactivity (23%) and impairment of prosocial behavior (5.7%).

The total problem score was more in 6-13 years age group in both genders in comparison with 14-18 years age group (Table 1). There was a significant association between age and subscale of emotion among girls (p=0.003). Emotional problems were more prevalent in adolescent girls. The correlation between age and hyperactivity was more prevalent in both sexes and in lower age group (p=0.023 in girls, p=0.021 in boys). There was a significant association between age and peers` problems in 6-13 years old girls and it was more than 14-18 years girls old (p=0.04). Subscale of impact scale that showed total impairment was abnormal in 34.3% of samples and significant in both sexes (p=0.02) (Table 1).

**Table 1:** Frequency distribution (%) of five domains of SDQ based on sex and age groups of youth population.

		** Boys No. (%)**			** Girls No. (%)**		
**Age (Year)**		**6-10**	**11-13**	**14-18**	**6-10**	**11-13**	**14-18**
Emotional	Normal	246 (67.6)	170 (65.5)	251 (68.2)	232 (67.4)	156 (67.2)	217 (55.6)
	Borderline	46 (12.6)	25 (9.7)	39 (10.6)	29 (8.4)	15 (6.5)	51 (13.1)
	Abnormal	72 (19.8)	64 (24.7)	78 (21.2)	83 (24.1)	61 (26.3)	122 (31.3)
*P*-value			0.517			0.003	
Conduct	Normal	158 (43.4)	105 (40.9)	169 (45.4)	190 (56.2)	137 (58.5)	207 (53.6)
	Borderline	52 (14.3)	42 (16.3)	57 (15.3)	47 (13.9)	30 (12.8)	61 (15.8)
	Abnormal	154 (42.3)	110 (42.8)	146 (39.2)	101 (29.9)	67 (2 8.6)	118 (30.6)
*P*-value			0.779			0.768	
Hyperactivity	Normal	191 (52.5)	151 (58.5)	233 (63.8)	214 (63.1)	169 (72.8)	276 (71.7)
	Borderline	56 (15.4)	29 (11.2)	47 (12.9)	44 (13.0)	23 (9.9)	50 (13.0)
	Abnormal	117 (32.1)	78 (30.2)	85 (23.3)	81 (23.9)	40 (17.2)	29 (15.3)
*P*-value			0.021			0.023	
Peer	Normal	204 (56.0)	146 (58.2)	216 (59.3)	188 (54.8)	135 (58.2)	230 (59.6)
	Borderline	54 (14.8)	36 (14.3)	66 (18.1)	52 (15.2)	30 (12.9)	73 (18.9)
	Abnormal	106 (29.1)	69 (27.5)	82 (22.5)	103 (30.0)	67 (28.9)	83 (21.5)
*P*-value			0.254			0.04	
Total	Normal	171 (48.0)	119 (49.6)	194 (56.1)	195 (58.9)	137 (61.2)	225 (61.8)
	Borderline	73 (20.5)	45 (18.8)	66 (19.1)	45 (13.6)	31 (13.8)	47 (12.9)
	Abnormal	112 (31.5)	76 (31.7)	86 (24.9)	91 (27.5)	56 (25.0)	92 (25.3)
*P*-value	p-value		0.195			0.938	
proSocial	Normal	315 (86.8)	222 (85.7)	312 (84.3)	309 (91.2)	218 (92.8)	352 (89.8)
	Borderline	26 (7.2)	18 (6.9)	25 (6.8)	18 (5.3)	10 (4.3)	21 (5.4)
	Abnormal	22 (6.1)	19 (7.3)	33 (8.9)	12 (3.5)	7 (3.0)	19 (4.8)
*P*-value			0.701			0.724	
Impact	Normal	78 (56.9)	44 (44.4)	70 (42.7)	83 (59.7)	44 (52.4)	62 (40.0)
	Borderline	24 (17.5)	13 (13.2)	26 (15.9)	26 (18.7)	16 (19.0)	26 (16.8)
	Abnormal	35 (25.5)	42 (42.4)	68 (41.5)	30 (21.6)	24 (28.6)	67 (43.2)
*P*-value			0.029			0.02	

Excluding sex, the chance of abnormal total problem score in 6-10 years and 11-13 years age groups was 2.39 folds (OR: 2.39 , 95% CI: 1.52-3.765) and 2.17 folds (CI=1.31-3.59, OR: 2.17, 95%) of 14-18 years age group respectively (Table 2).

**Table 2:** Multinominal logistic regression results of some effective factors on SDQ total problem scores.

**Characteristics**	****	**Borderline**	**Abnormal**
****		OR(95%CI)	OR (95%CI)
Age (Year)	6-10	1.48 (0.91-2.42)	2.39 (1.52-3.76)^a^
****	11-13	1.47 (0.85-2.55)	2.17 (1.31-3.59)^a^
****	14-18	1=Reference	1=Reference
Father education	Primary or less	1.17 (0.55-2.49)	2.61 (1.32-5.16)^a^
	Secondary or high school	1.34 (0.72-2.51)	2.59 (1.44-4.65)^a^
	College	1.15 (0.66-1.99)	1.76 (1.04-2.97)^a^
	University or high	1=Reference	1=Reference
Mother job	Employee	0.41 (0.21-0.79)	0.41 (0.22-0.77)
	Housekeeper	1=Reference	1=Reference
Sex	Male	1.82 (1.19-2.78)a	1.11 (0.76-1.64)
	Female	1=Reference	1=Reference
Social	Normal	0.38 (0.14-1.06)	0.17 (0.07-0.42)
	Borderline	0.44 (0.12-1.67)	0.44 (0.14-1.38)
	Abnormal	1=Reference	1=Reference
Impact	Normal	0.26 (0.16-0.44)a	0.11 (0.07-0.17)^a^
	Borderline	0.61 (0.32-1.18)	0.32 (0.18-0.57)^a^
****	Abnormal	1=Reference	1=Reference

^a^ Statistically significant

In regard to parents` demographic features, psychiatric problems in children with housekeeper mother was 2.44 folds more than working mothers` (CI: 0.22-0.77, OR: 0.41, 95%). The chance of psychiatric disorders in children with illiterate or low literate fathers was 2.6 folds when compared to those with highly educated ones (Table 2). The findings showed that having abnormal score of prosocial behavior and impact score may increase the chance of psychiatric disorders by 6 and 9.1 folds respectively (Table 2).

## Discussion

It was shown that 26% of youths had an abnormal total problem score while this rate in a study carried out in Tehran was 25.8% in children and 13.7% in adolescents when they used the same research tools and method.[17] In other studies conducted on children in Brazil and Gaza strip by using SDQ, the frequency of total problems was 18.7%[18] and 14.2% respectively.[19] The differences may be due to variation in residence area, culture and educational level.

In this study, conduct disorder had the highest prevalence (34.7%) which is consistent with Mohammadi et al. studies[17][20] but is not consistent with those of Brazil,[18] Gaza[19] and Siberia[9] that reported emotional problems as the most prevalent disorder. This discrepancy needs to be studied.

This study showed a significant association between age and emotional disorders in girls aged 13-18 years which is consistent with Alavi et al. study.[21] Since girls used more internalizing mechanisms than boys to face the problems,[22] emotional problems (depression and anxiety) were more prevalent among them. There was significant association between age and subscale of hyperactivity at lower ages that is consistent with Mohammadi et al.[20] and Solbodskaya et al. studies[9] in which young age was named as a specific factor for externalizing behaviors. In this study, the association between age and peers problems in girls was significant and the problems were less at older ages.

Gavin et al. reported that the relationship of girls with peers group was more positive. They also reported less negative behavior in peers group in late adolescents when compared with early and middle age groups.[23] This study revealed that impact score had a significant association with age, therefore at higher ages, psychiatric problems caused more personal distress and impairment. These results were not consistent with Widenfelts et al. study.[24]

The chance of abnormal scores of total problems in youths whose mothers were housekeepers was 2.5 folds more than those with working mothers. In a study in 2007 in Isfahan, the highest prevalence of behavioral disorders was related to youths whose mothers were housekeepers.[13] Employee mothers generally had a better family economic status and could reduce depressive signs and finally affect children's mental health.

Results showed that abnormal total problems in youths were related to fathers with lower level of education. Farshidnejad et al. also reported that high educated fathers had children with less behavioral disorders.[13] The chance of abnormal total problems in youths who had abnormal prosocial score was expected to be higher. It shows that youths who had more problems had less socially acceptable behavior. However, gathering information from adolescents and parents about emotional and behavioral problems is crucial for diagnosis.[25]

The use of one questionnaire in the present study could be a limitation and the method of home referring to pick up the answers can be another limitation in this study. Our findings showed that mental health of youth population in Isfahan especially the conduct problem needs more attention.

## References

[1] De Silva MJ, McKenzie K, Harpham T, Huttly SR (2005). Social capital and mental illness:a systematic review.. J Epidemiol Community Health.

[2] Nikapota AD (1991). Child psychiatry in developing countries.. Br J Psychiatry.

[3] Simpson GA, Bloom B, Cohen RA, Blumberg S, Bourdon KH (2005). U.S. children with emotional and behavioral difficulties: data from the 2001, 2002, and 2003 National Health Interview Surveys.. Adv Data.

[4] Smit F, Cuijpers P, Oostenbrink J, Batelaan N, De Graaf R, Beekman A (2009). Costs of nine common mental disorders: Implications for curative and preventive psychiatry.. J Ment Health Policy Econ.

[5] Ford T, Goodman R, Meltzer H (2003). The British Child and Adolescent Mental Health Survey 1999: the prevalence of DSM-IV disorders.. J Am Acad Child Adolesc Psychiatry.

[6] Offord DR, Boyle MH, Fleming JE, Blum HM, Grant NI (1989). Ontario Child Health Study. Summary of selected results.. Can J Psychiatry.

[7] Davies S, Heyman I, Goodman R (2003). A population survey of mental health problems in children with epilepsy.. Dev Med Child Neurol.

[8] Goodman R, Scott S (2005). Child psychiatry. 2nd edition.

[9] Slobodskaya HR, Akhmetova OA, Ryabichenko TI (2007). Siberian child and adolescent mental health: prevalence estimates and psychosocial factors.. Alaska Med.

[10] Rønning JA, Handegaard BH, Sourander A, Mørch WT (2004). The Strengths and Difficulties Self-Report Questionnaire as a screening instrument in Norwegian community samples.. Eur Child Adolesc Psychiatry.

[11] Mohammadi MR, Davidian H, Noorbala AA, Malekafzali H, Naghavi HR, Pouretemad HR, Yazdi SA, Rahgozar M, Alaghebandrad J, Amini H, Razzaghi EM, Mesgarpour B, Soori H, Mohammadi M, Ghanizadeh A (2005). An epidemiological survey of psychiatric disorders in Iran.. Clin Pract Epidemiol Ment Health.

[12] Hosseini Fard SM, Birashk B, Atef VM (2005). Prevalence of mental disorders in high school students in Rafsanjan.. Andisheh Va Raftar.

[13] Farshidnejad A (2004). Prevalence of behavioral disorder in primary schools of Isfahan.. Behavioral Sciences Research Journal.

[14] Ganizadeh A, Izadpanah A, Abdollahi G (2007). Scale validation of the strengths and difficulties questionnaire in Iranian children.. Iran J Psychiatry.

[15] Goodman R (2001). Psychometric properties of the strengths and difficulties questionnaire.. J Am Acad Child Adolesc Psychiatry.

[16] Goodman R, Meltzer H, Bailey V (2003). The Strengths and Difficulties Questionnaire: a pilot study on the validity of the self-report version.. Int Rev Psychiatry.

[17] Mohammdi MR, Mahmoudi-Gharaei J, Tehrani-Doost M, Shahrivar Z (2008). Prevalence of psychiatric disorders among adolescents in Tehran.. Iranian Journal of Psychiatry.

[18] Cury CR, Golfeto JH (2003). Strengths and difficulties questionnaire (SDQ): a study of school children in Ribeirão Preto.. Rev Bras Psiquiatr.

[19] Thabet AA, Stretch D, Vostanis P (2000). Child mental health problems in Arab children: application of the strengths and difficulties questionnaire.. Int J Soc Psychiatry.

[20] Mohammdi MR, Mahmoudi-Gharaei J, Tehrani-Doost M, Shahrivar Z, Saadat S (2008). Children mental health problems: parent report form of strengths and difficulties questionnaire.. Iranian Journal of Psychiatry.

[21] Alavi A, Mohammadi MR, Mahmoudi-Gharaei J, Tehrani-Doost M, Shahrivar Z (2009). The farsi version the strengths and difficulties questionnaire self-report form: the normatative data and scale properties.. Iran J Child Neurology.

[22] Leadbeater BJ, Kuperminc GP, Blatt SJ, Hertzog C (1999). A multivariate model of gender differences in adolescents' internalizing and externalizing problems.. Dev Psychol.

[23] Gavin LA, Furman W (1986). Age differences in adolescents` perceptions of their peer group.. Development Psychology.

[24] van Widenfelt BM, Goedhart AW, Treffers PD, Goodman R (2003). Dutch version of the Strengths and Difficulties Questionnaire (SDQ).. Eur Child Adolesc Psychiatry.

[25] Van Roy B, Groholt B, Heyerdahl S, Clench-Aas J (2010). Understanding discrepancies in parent-child reporting of emotional and behavioural problems: Effects of relational and sociodemographic factors.. BMC Psychiatry.

